# Supplementary figures and images for: Identification and validation of shared gene signature of kidney renal clear cell carcinoma and COVID-19
Source: PeerJ. 2024 Mar 4;12:e16927. doi: 10.7717/peerj.16927 (PMC10921934; doi:10.7717/peerj.16927)

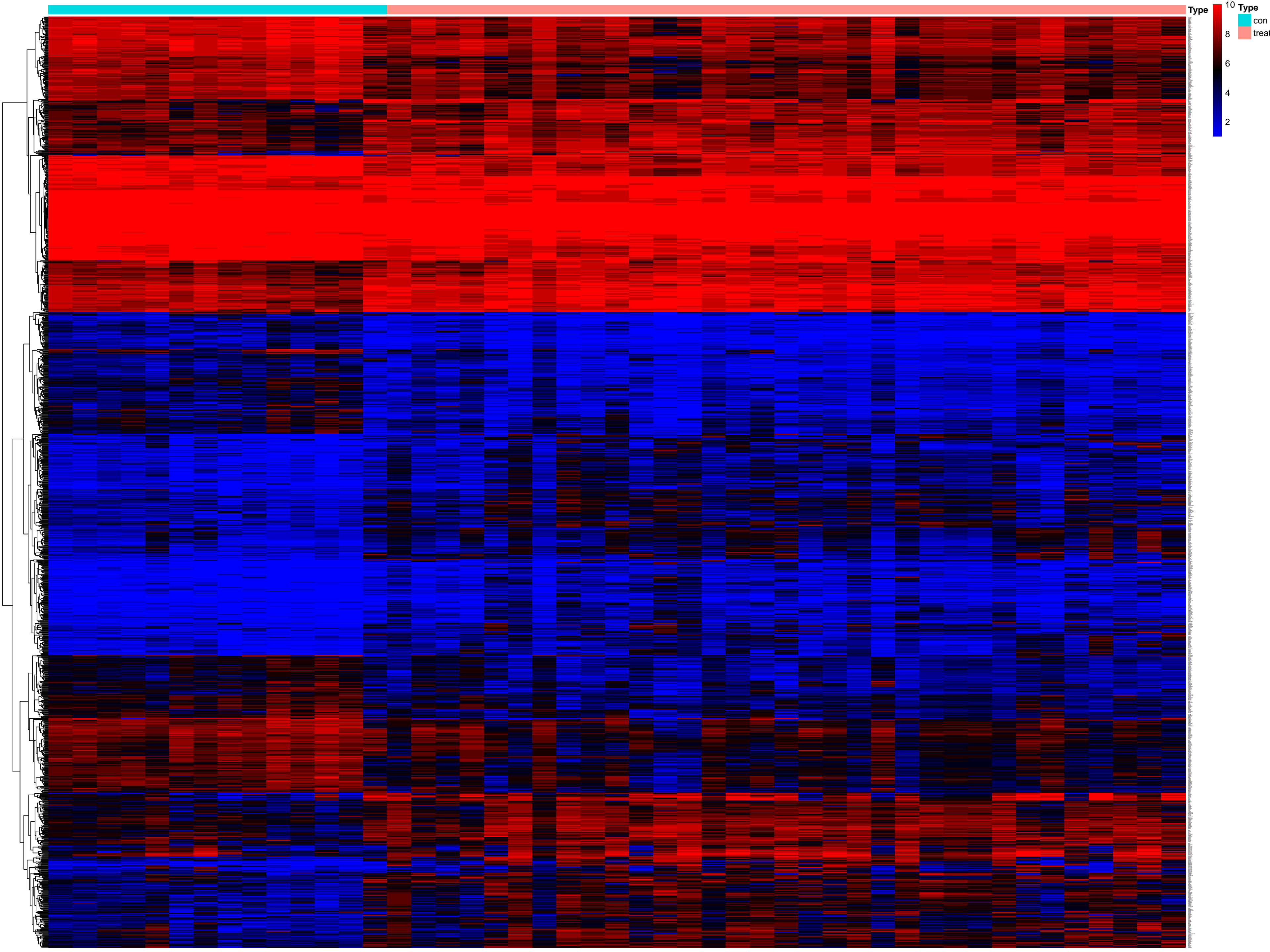

Supplement: Supplemental Information 2 [file peerj-12-16927-s002.pdf]

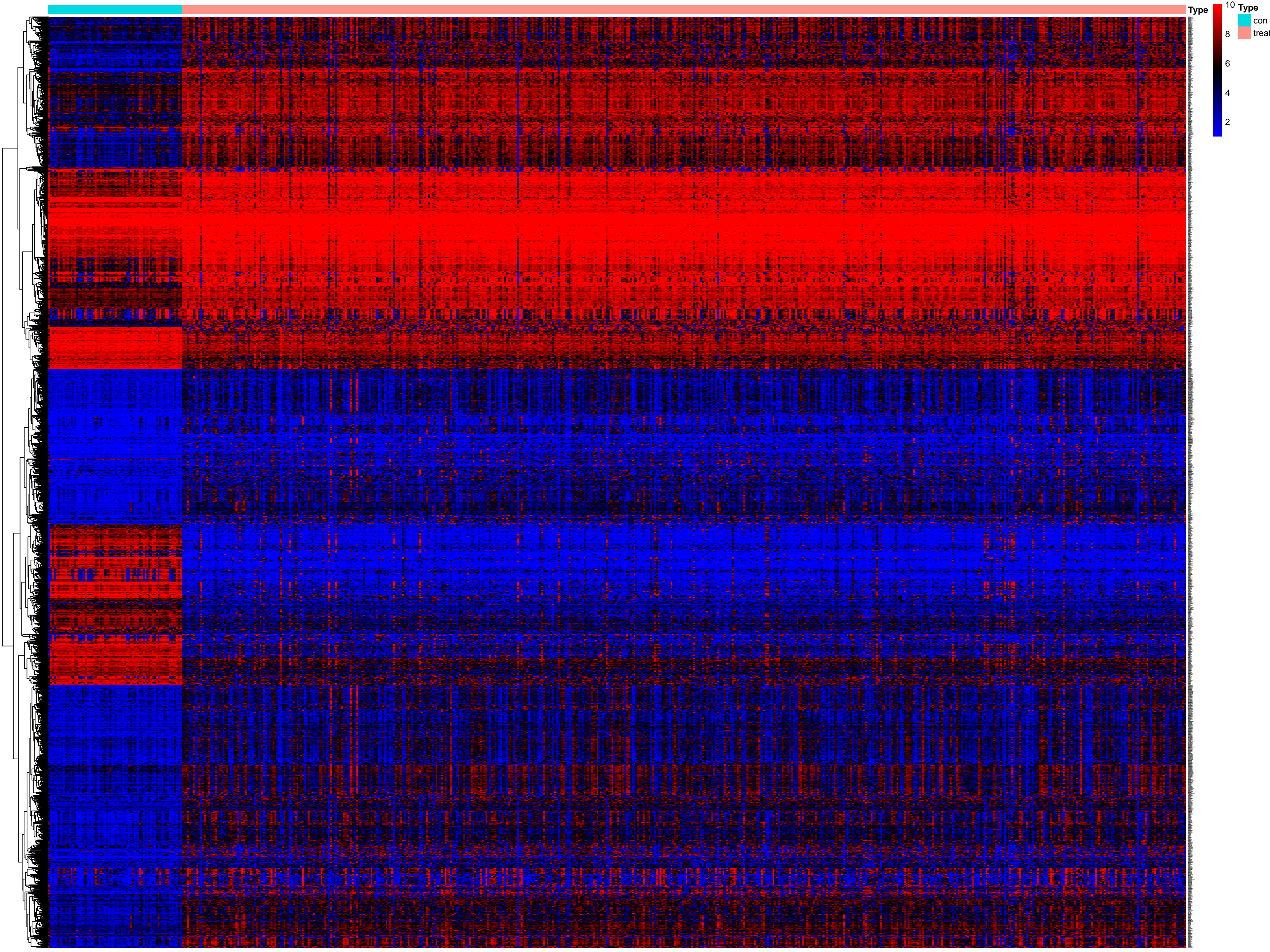

Supplement: Supplemental Information 3 [file peerj-12-16927-s003.pdf]

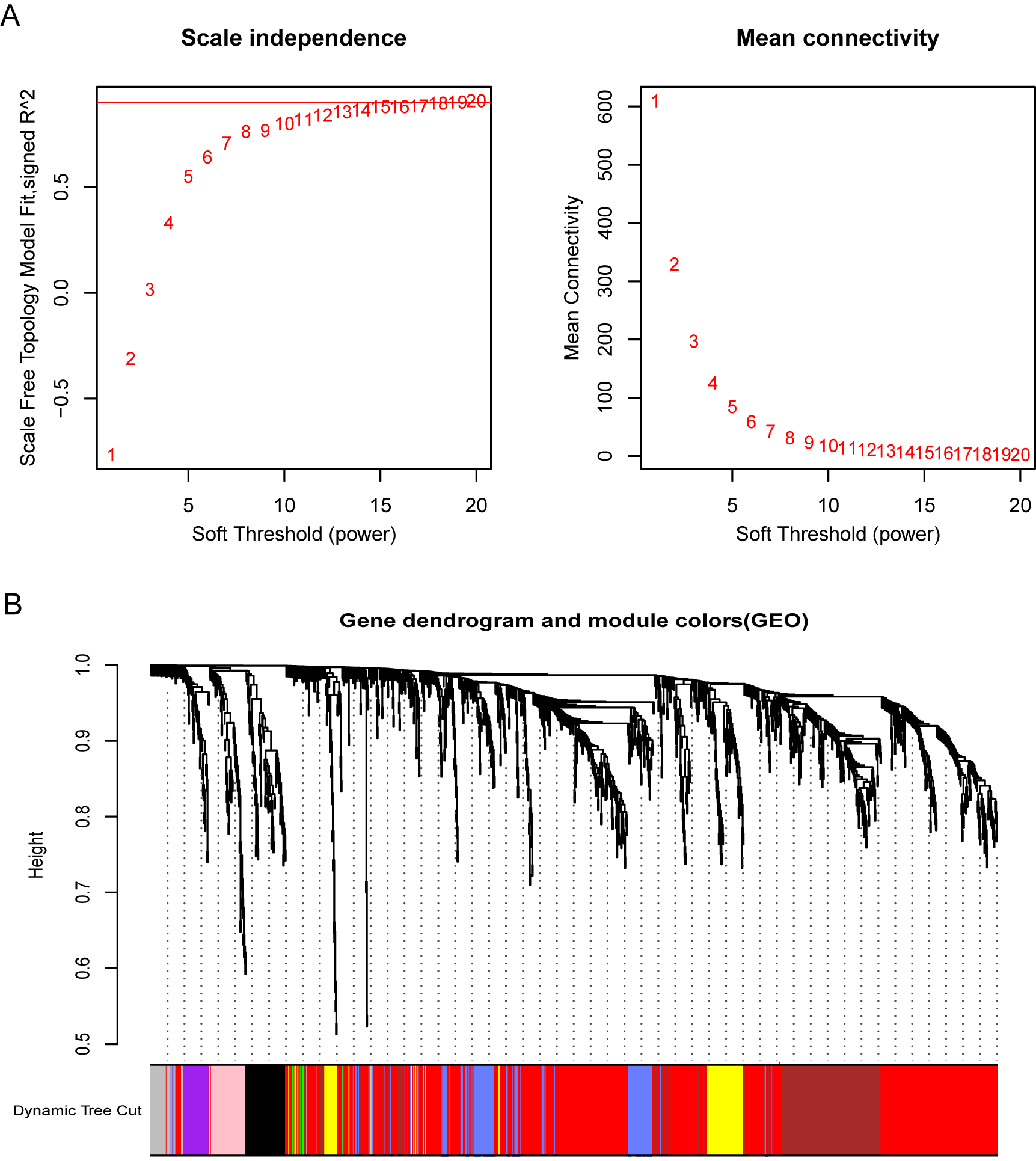

Supplement: Supplemental Information 4 — (A) Demonstration of scale independence and mean connectivity of WGCNA, choosing a soft threshold of 13 and a scale-free topological fit index of 0.9 resulted in relatively balanced scale independence and mean connectivity of WGCNA; (B) Systematic tree diagram and trait correlation heat map of genes. [file peerj-12-16927-s004.png]

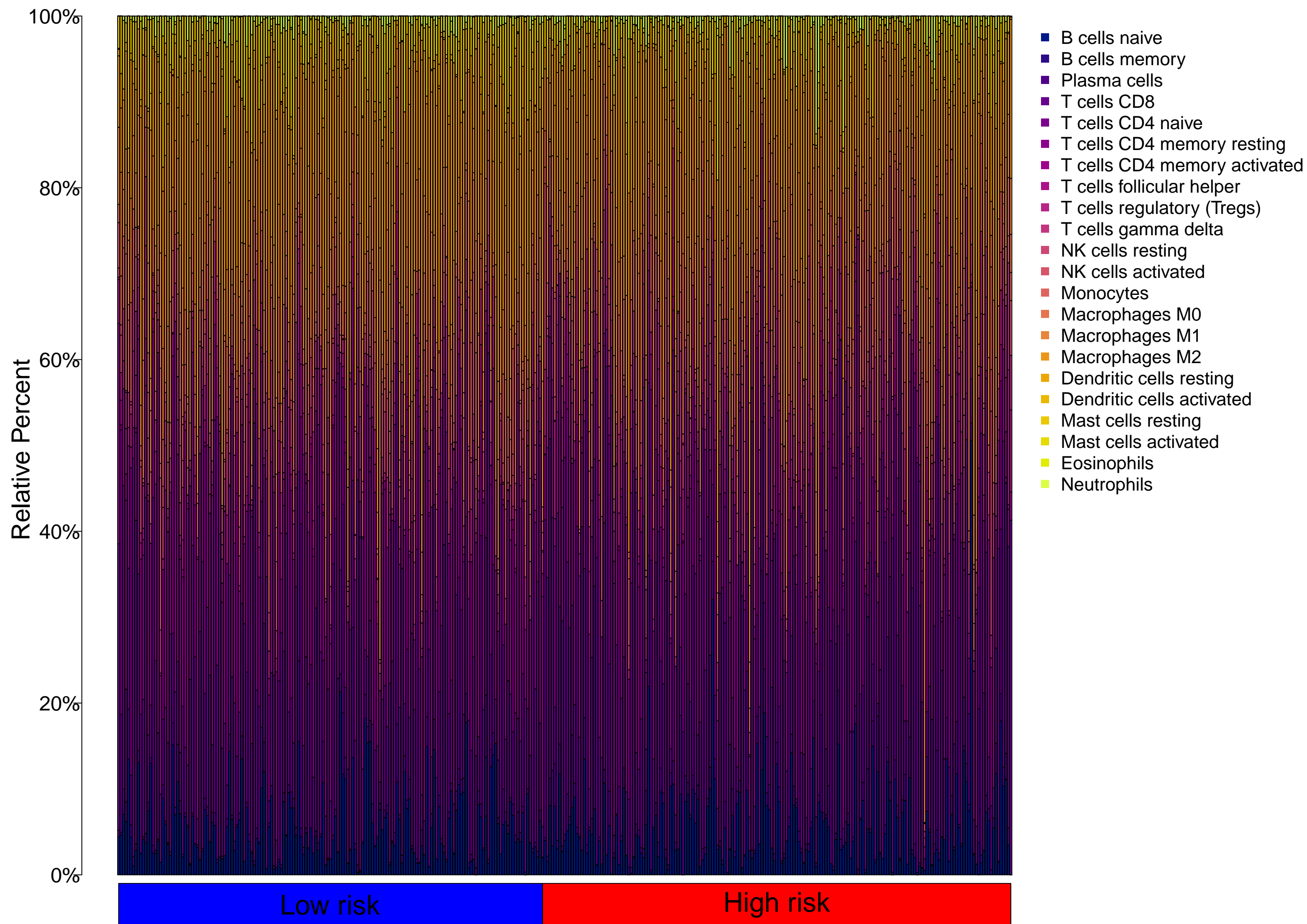

Supplement: Supplemental Information 5 [file peerj-12-16927-s005.pdf]

1 CEACAM4


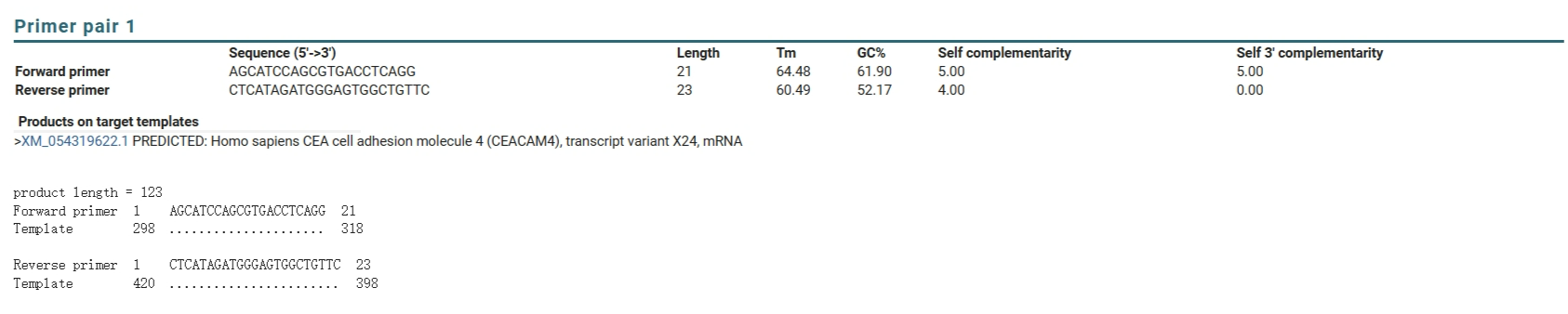


2 HECW2


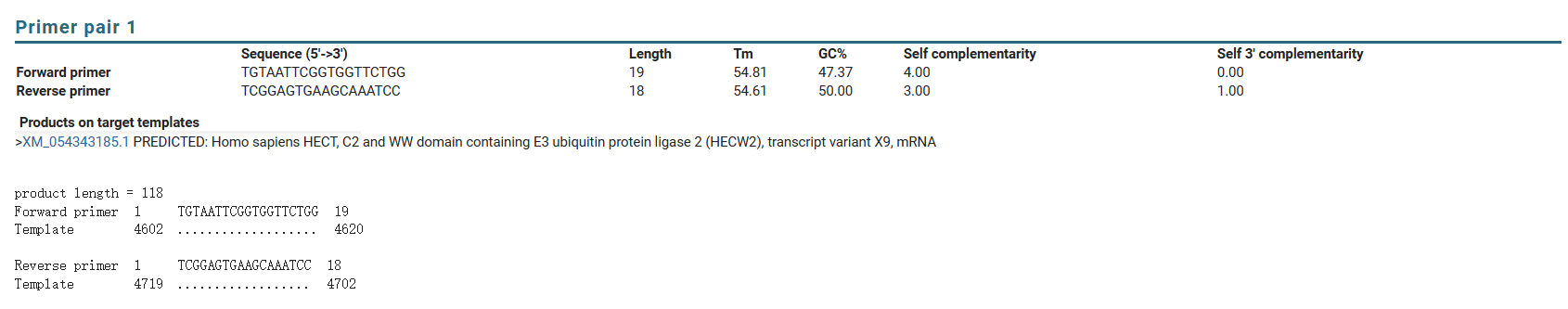


3 GTSE1


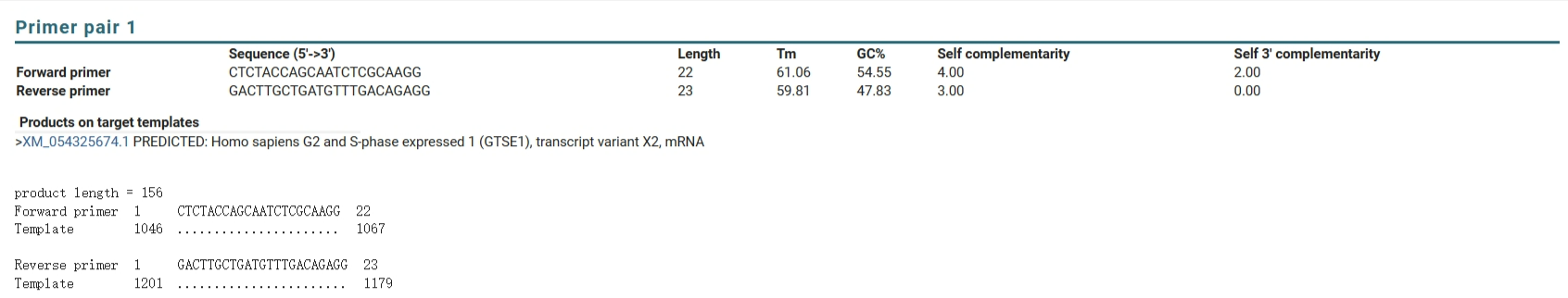


4 KCNMA1


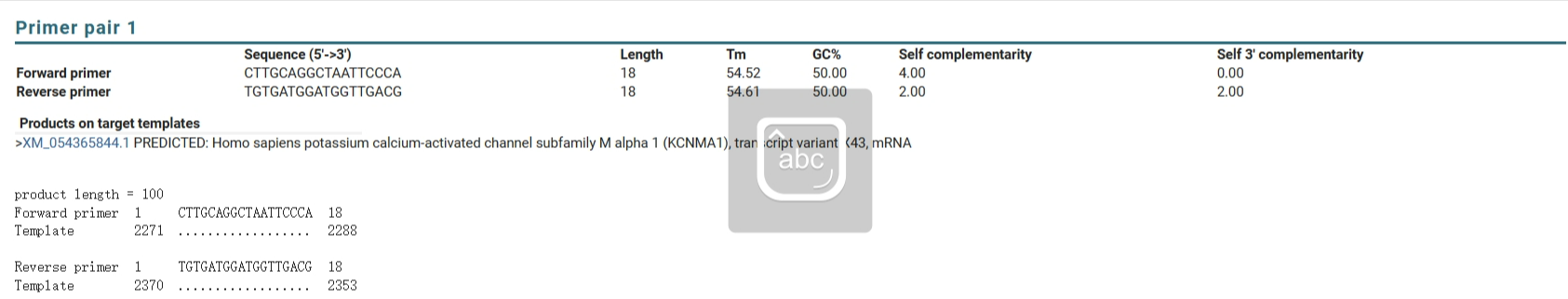

Supplement: Supplemental Information 11 [file peerj-12-16927-s011.docx]
